# Supplementary material for: Getting robots back on track by reconstituting control in unexpected situations with online learning
Source: Nat Commun. 2026 Mar 9;17:3715. doi: 10.1038/s41467-026-70256-y (PMC13102915; doi:10.1038/s41467-026-70256-y)
Supplement: Supplementary file 1 — Supplementary Information [file 41467_2026_70256_MOESM1_ESM.pdf]

Getting Robots Back On Track by Reconstituting Control in Unexpected Situations with Online Learning

Supplementary Related Work

Model-predictive Control

Many prior methods tackling the problem of adaptation to perturbations have been concerned with designing new controllers or policies that aim to maintain operability under a pre-specified set of perturbations. For example, conventional control methods involve physically modelling the system and performing trajectory optimisation [1] and fast re-planning through model-predictive control approaches [2–4]. To handle perturbations such as slippage or contact forces, these methods commonly explicitly model and estimate these perturbation events as well as the corresponding reaction required from controllers to dictate switches for a finite state machine [5, 6]. However, such approaches quickly increase in complexity when more sources of perturbation are considered and the designed models might become worse if the robot operates in a scenario that hasn't been considered during training time [7].

Reinforcement Learning

On the other hand, Reinforcement Learning (RL) [8] has recently gained popularity as it aims to simplify the manual design process of controllers, and can result in controllers that demonstrate behaviours that cannot be modelled, designed or engineered [9–11]. Being a data-driven approach, RL requires controllers to be optimised beforehand using a large amount of interaction with the environment under different perturbations to ensure the controller remains operable when facing them. This is done through domain randomisation in simulation, where a specified range of values from a wide variety of factors such as friction, restitution, robot mass, and more can be varied at each roll-out [12–15]. Additionally, RL frequently requires long learning times and complex data generation processes through large-scale simulation [16, 17].

Some research directions in RL are closely related to our work, such as Continual RL [18, 19], Meta-RL [20–22], and Residual RL [23, 24]. However, these approaches differ from the motivation behind FLAIR in several important aspects. First, these methods typically aim to learn a full controller that can be directly applied to the robot, without relying on an existing low-level controller or on human inputs. FLAIR

adopts a different perspective by introducing a hierarchical layer applied on top of an existing low-level controller rather than replacing it, and adapting incoming human commands. Second, most of these approaches require several seconds to minutes of data on the new task to perform adaptation. A key property of FLAIR is that it adapts in less than a second, from only a few datapoints.

To illustrate the limitations of such data-driven fine-tuning methods in real online scenarios, we include a Residual RL baseline in our experiments (described in detail in the Baselines Section below). This baseline uses the same data processing pipeline and hardware as FLAIR, but learns a residual controller online using RL on the robot. In our experiments, this led to unsafe behaviors such as sudden accelerations or sudden backward movements, requiring the trials to be stopped. This result illustrates the challenges of applying online residual RL in the presence of perturbations and clarifies the conditions under which FLAIR provides reliable adaptation.

More generally, FLAIR is designed to complement any low-level controller, including RL-trained controllers. Its hierarchical structure allows it to be integrated into RL-based pipelines, where it can be applied at inference time as an online adaptation mechanism that adjusts operator inputs. Recent work has also highlighted the difficulties faced by gradient-based learning approaches, including RL, in continual or lifelong learning settings [25]. FLAIR offers an alternative direction for achieving rapid adaptation to perturbations without relying on continual gradient updates.

Online Learning

Both these families of approaches rely on designing new controllers to handle any additional type of perturbation. Most similar to our approaches are online learning methods which adapt while collecting data using trials during deployment [26–29]. Self-modelling approaches [26, 27] refine a model of a robot's topology and morphology in simulation to match data collected using online interactions. Alternatively, Cully et al. [29] learn a diversity of priors and skills in simulation [30, 31] that function as redundancies for rapid online adaptation to mechanical damage. However, these methods require minutes [29, 32, 33] to hours or days [26] to adapt. Nygaard et al. [28] adapt the morphology of a robot to overcome terrain changes, but require morphology-adaptable robots and do not consider perturbations.

Fault-Tolerant Control

As another line of work, Fault-tolerant control has become a major field of research that tries to deal with adaptation in the situation of damage or a disturbance by focusing on Fault Detection of Sensors and Fault Recovery once the faulty sensors have been identified and isolated [34]. Mathematical models are learned to generate residual signals that can be used to detect a fault and learn a controller to recover them [35]. The concept of fault-tolerant control can even be applied to a fleet of mobile robots [36]. Fault-Tolerant Control can be extended with the concepts of Active Inference for Robot Control [37, 38]. Active inference follows the principle of free-energy (or surprise) minimisation between observations and actions. In active inference, the believed state of the robot is used by two generative models to predict the true sensory

data and dynamics of the robot. Based on this estimated state, a controller input can be calculated with gradient descent to reach the desired state [37, 39]. More recent work presents the relationship between Active Inference controllers and PID controllers [40] or the unbiased Active Inference controller, an improvement over Active Inference controllers, which can be applied to a real 7-DoF robotic arm [41]. The biggest difference to this line of work is the estimation of faults, which is a key factor for fault-tolerant control, whereas FLAIR does not diagnose faults in the system but learns the impact of perturbations on its behaviour. It then adjusts the commands to follow a desired behaviour. Our approach assumes the sensors are noisy but not faulty.

Adaptive Control

A lot of recent methods for drone control have started incorporating complex non-linear dynamics models as Deep Neural Networks, to learn higher-order interactions between different inputs, resulting in better control and stability guarantees [7, 42]. The authors of these methods implement a non-linear feedback controller as a baseline [43], similarly to our $\mathcal{L}_1$ baseline. In contrast to adaptive control, FLAIR has the possibility to model the non-linear impact of the perturbations on the behaviours of the robot, which makes it adapt quicker by hierarchically decoupling global and local adaptations.

Supplementary Baselines

$\mathcal{L}_1$ Adaptive Control

For our first baseline, we use a state-of-the-art adaptive controller used in drone applications, namely the $\mathcal{L}_1$ Adaptive Control method [7, 44]. $\mathcal{L}_1$ Adaptive Control has been designed to offer a robust controller against perturbations by learning the disturbances at every time-step. We follow the implementation steps from Hovakimyan et al. [44]. Our robot is directly controlled with speed commands $\mathbf{a} = (v_x, \omega_z)$ which means that we are searching for the optimal commands to reach a certain behaviour $\mathbf{b}' = (v'_x, \omega'_z)$ corresponding to the velocities of the robot. The reference model can be described as follows:

$$\mathbf{b}' = \mathbf{A}\mathbf{b} + \mathbf{B}\mathbf{a} + \mathbf{B}\sigma_{\text{hat}} \tag{1}$$

with

$$\mathbf{A} = \begin{bmatrix} -0.1 & 0 \\ 0 & -0.1 \end{bmatrix}$$

$$\mathbf{B} = \begin{bmatrix} 1 & 0 \\ 0 & 1 \end{bmatrix}$$

$$\mathbf{P} = \begin{bmatrix} 1 & 0 \\ 0 & 1 \end{bmatrix}$$

where $\mathbf{b}$ is the current velocity of the robot $\mathbf{b} = (v_x, \omega_z)$.

In addition to the linear system, $\mathcal{L}_1$ Adaptive Control uses a gain $\Gamma = 1$ to correct the predicted movement with the predicted disturbances:

$$\mathbf{e} = \mathbf{x} - \hat{\mathbf{x}} \tag{2}$$
$$\dot{\sigma}_{\text{hat}} = \Gamma \mathbf{P}\mathbf{e} \tag{3}$$
$$\sigma_{\text{hat}} \leftarrow \text{clip}(\dot{\sigma}_{\text{hat}}, -\sigma_{\text{max}}, \sigma_{\text{max}}) \tag{4}$$

This translates into the following $\mathcal{L}_1$ Adaptive Control Law to execute action a:

$$\mathbf{a}_{\text{ref}} = \mathbf{B}^{-1}(\mathbf{b}' - \mathbf{A}\mathbf{b}) \tag{5}$$
$$\mathbf{a}_{\text{ad}} = -\mathbf{C} \cdot \sigma_{\text{hat}} \tag{6}$$
$$\mathbf{a} = \text{clip}(\mathbf{a}_{\text{ref}} + \mathbf{a}_{\text{ad}}, \mathbf{a}_{\text{min}}, \mathbf{a}_{\text{max}}) \tag{7}$$

We tuned the parameters of the $\mathcal{L}_1$ controller on two tasks on the Chicane Track: 1) no perturbation and 2) a static perturbation (impaired track). The second task ensures the $\mathcal{L}_1$ controller performs effectively in terms of circuit completion time when applying perturbations. In this situation, $\mathcal{L}_1$ outperforms FLAIR, indicating that the parameters are appropriately tuned. Since the focus of the paper is on adaptation without prior information on the experienced condition, we fixed the $\mathcal{L}_1$ parameters and did not tune them to each individual track or perturbation.

Linear Quadratic Regulator (LQR)

As an additional baseline, we chose an optimal control algorithm that can run in real-time on our system. Since our system can be linearised around its operation point, we leverage the Linear Quadratic Regulator algorithm (LQR) [45–47]. LQR has been successfully used in various forms for different robotic problems, such as flying [48] or robotic locomotion [49–51]. We want our robot to closely follow the commands of the driver for the next time-step without planning more than one step ahead, which is why the use of methods such as Iterative LQR would not be appropriate [52]. Our robot is directly controlled with speed commands $\mathbf{a} = (v_x, \omega_z)$, which means that we are searching for the optimal commands to reach a certain behaviour $\mathbf{b}' = (v'_x, \omega'_z)$ corresponding to the velocities of the robot. This linear system can be described as:

$$\mathbf{b}' = \mathbf{A}\mathbf{b} + \mathbf{B}\mathbf{a} \tag{8}$$

with

$$\mathbf{A} = \begin{bmatrix} 0 & 0 \\ 0 & 0 \end{bmatrix}$$
$$\mathbf{B} = \begin{bmatrix} 1 & 0 \\ 0 & 1 \end{bmatrix}$$

where $\mathbf{b}$ is the current velocity of the robot $\mathbf{b} = (v_x, \omega_z)$.

In our case, we would like to control the velocity of the robot directly via the commands $\mathbf{a}$ which are velocity commands. This means that the matrix A is a zero

matrix, and the optimal command $\mathbf{a}*$ can be found by solving the continuous-time Riccati equation and getting the LQR gain matrix $\mathbf{K}$. To calculate $\mathbf{K}$, we set the cost matrices $\mathbf{Q}$ for the velocity error and $\mathbf{R}$ for the effort matrix with empirically found values:

$$\mathbf{Q} = \begin{bmatrix} 0.3 & 0 \\ 0 & 0.08 \end{bmatrix}$$

$$\mathbf{R} = \begin{bmatrix} 1 & 0 \\ 0 & 1 \end{bmatrix}$$

Solving the Riccati equations, we can get the optimal commands $\mathbf{a}*$:

$$\begin{bmatrix} error_{v_x} \\ error_{\omega_z} \end{bmatrix} = \mathbf{b} - \mathbf{a}$$

$$\mathbf{a}* = \mathbf{a} - (\mathbf{K} \begin{bmatrix} error_{v_x} \\ error_{\omega_z} \end{bmatrix}) \tag{9}$$

Equation 9 will return the optimal commands $\mathbf{a}*$ to execute on the robot in order to achieve the desired command $\mathbf{a}$ (reference commands). In comparison to the iterative-LQR algorithm, LQR can be solved quickly and in real-time on the robot to find the best actions and can correct any deviations from the user's commands to counter perturbations such as damage or slipping. We solve Equation 9 at every time-step t.

Reinforcement Learning (RL)

We also demonstrate the advantages of our proposed learning approach in comparison to a reinforcement learning (RL)-based method. We consider a Markov Decision Process (MDP) where at each timestep t, an agent in state $\mathbf{s_t}$ takes an action a_t that leads it into a new state $\mathbf{s_{t+1}}$ determined by the transition-probability $T(\mathbf{s_t}, \mathbf{a_t}, \mathbf{s_{t+1}})$. Each such transition leads to a reward r_t given by a function $R(\mathbf{s_t}, \mathbf{a_t})$. The RL problem aims to learn a policy π for the agent that maximises the total reward over an episode of length T: $\mathbb{E}_\pi[\sum_{t=0}^{T-1} r_t]$ [8]. In actor-critic RL approaches, as used here, the policy π is represented as a neural network known as the actor, and optimised via gradient ascent to maximise the action-state value function $Q(\mathbf{s_t}, \mathbf{a_t}) = \mathbb{E}_\pi[\sum_{k=0}^{T-t} \gamma^k r_{t+k+1} \mid \mathbf{s_t}, \mathbf{a_t}]$, approximated using a neural network known as the critic. Here, we use the Twin Delayed Deep Deterministic policy-gradient algorithm (TD3) algorithm [53], a widely recognised actor-critic algorithm in the field. TD3 uses two critic networks Q_{θ_1} and Q_{θ_2}, with the same structure but trained separately to counteract overestimations, and learns a deterministic actor network. TD3 also uses target networks that are updated with a delay to provide further learning stability.

To ensure fairness in the comparison, we do not use pre-training, enabling the RL agent to learn fully online and directly from sensor data, similarly to our FLAIR approach. To mitigate the complexity of this setup, we adopt residual RL [23, 24], where the RL agent learns only the differential command applied atop the human intent, rather than learning the full command. For consistency, we employ the exact same data-processing pipeline as used for FLAIR. The reward function for the RL algorithm is defined as the negative distance between the user's intent and the

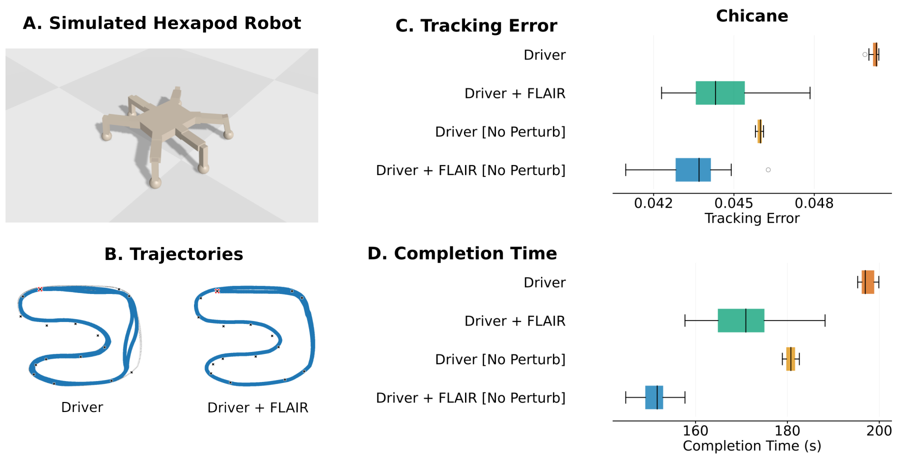

Fig. 1 FLAIR's Performance on the Hexapod task. We compare FLAIR against baselines on the (A) Simulated Hexapod robot, following (B) a chicane track, where we give some example trajectories of "Driver" and "Driver + FLAIR". We show the benefits of FLAIR in (C) Tracking Error and (D) circuit Completion Time. In the box-plots, the center line represents the median, the box limits denote the upper and lower quartiles, the whiskers extend to 1.5x the interquartile range, and individual points represent outliers.

corresponding effect observed in sensor readings, as aligned by our data collection pipeline.

Our RL implementation is based on the CleanRL framework [54], specifically utilising the JAX implementation of the TD3 algorithm. This implementation facilitates sufficiently fast training, achieving multiple model updates per minute. To adapt TD3 for the online setting, we retain all hyper-parameters from the original paper, except for two modifications: the network size is halved to simplify learning in the online regime, and the network initialization is adjusted to initialize the residual to zero by setting the final layer weights to zero, following prior work [23].

When evaluating this baseline on our circuits, it frequently exhibited unsafe behaviour, often causing the robot to move in the opposite direction of the target. To address this, we established a safety condition: any run in which the distance to the next target increased by more than 1 metre over time (meaning the controller is moving away from the target) was terminated. Under these conditions, the proposed RL baseline failed to complete any complete runs on the Chicane circuit, whereas none of the other considered algorithms required termination. For safety reasons, we did not test it on more complex circuits. RL methods are known for requiring large quantities of data to learn meaningful actions. We hypothesise that the combination of a low-data regime and the highly noisy conditions of our experimental setup was particularly detrimental to this approach, severely limiting its effectiveness.

Supplementary Hexapod Results

To demonstrate the generalisation of FLAIR, we provide additional results on a simulated Hexapod pipeline, detailed below.

Experimental Setup

We consider a 6-legs Hexapod robot with 18-degrees-of-freedom, illustrated in Figure 1.A, similar to the one used in Cully et al. [29]. We use the Brax simulator [17] written in Jax [55] to simulate the robot. We drive our Hexapod robot around a Chicane track similar to the one used in the main experiments and illustrated in Figure 1.B. To do so, we use the same waypoint-tracking automatic driver as for the GVR-Bot in the main experiments. For perturbations, we consider a loss in the strength of the actuators of the right middle leg of the robot. We apply this perturbation directly to the model of the robot in the simulator.

Low-Level Controllers

To control the Hexapod robot, we use sinusoidal open-loop controllers within each of the motors. Each controller outputs position-control for its corresponding motor following a sinusoidal signal. Each controller also differs from the others in amplitude and phase of the sinusoid, enabling the emergence of complex movements. Following Cully et al. [29], for stability of the gaits, we apply the same sinusoid in the middle and bottom motor of each leg, but with negative amplitude.

We use a Quality-Diversity algorithm [31], MAP-Elites [56], in order to generate low-level controllers for a wide variety of linear velocity and angular velocity commands. In other words, we use MAP-Elites to enable controlling the hexapod robot similarly to a differential drive robot. In the following, when the driver requests a specific command, the corresponding set of sinusoidal controllers for every motor of the robot is loaded from the population and applied to the hexapod.

Results

The results for the simulated Hexapod task are provided in Figure 1 and Table 1. We also provide the p-value for significance in Table 2.

Overall, FLAIR reconstitutes 138.8% of operability in face of the perturbation, showcasing its good performance on this additional domain. Interestingly, the results show that FLAIR helps the driver recover operability even when no perturbation is applied. This result indicates that the low-level controllers found by MAP-Elites are not perfectly implementing the velocities requested by the driver. We hypothesize that this is due to two causes. First, the Brax simulator is intrinsically stochastic, leading to controllers behaving slightly differently during the training of MAP-Elites and during their usage by the driver. Second, during MAP-Elites training, controllers are evaluated from a standard reset position where all joints are set to their default position, while the driver chains controllers one after another. These differences might explain why FLAIR manages to reach even lower Tracking Error than the standard Driver by compensating for their effects. They can be seen as naturally occurring perturbations that are part of the environment, for which FLAIR successfully compensates.

		Chicane Broken Leg
(A) Tracking Error increase	Driver	9.4%
	Driver + FLAIR	-3.7%
	Driver + FLAIR [No Perturb]	-5.0%
(B) Completion Time increase	Driver	9.0%
	Driver + FLAIR	-5.4%
	Driver + FLAIR [No Perturb]	-16.1%
(C) FLAIR Tracking Error Reduction		138.8%
(D) FLAIR Completion Time Reduction		160.5%

Table 1 Summary of FLAIR's Performances on the Hexapod task. (A) Overhead with respect to the "Driver [No Perturbation]" baseline in terms of Tracking Error, and (B) in terms of Completion time. (C) We also quantify the Tracking Error Reduction by FLAIR as the reduction in increased Tracking Error for "Driver + FLAIR" with respect to the "Driver" (100% means the overhead in time or error is gone), and (D) in Completion Time. For all metrics, we compare the medians over 20 replications.

	Chicane Broken Leg
Tracking Error Driver	$6.3e-08$
Completion Time Driver	$6.3e-08$

Table 2 Statistical analysis. p-values computed using Wilcoxon Rank test for the comparison of the "Driver" and "Driver + FLAIR" results for the Hexapod.

Supplementary Boundary Analysis

The learned command-behaviour mapping is designed for systems based on differential-drive, given the focus of the paper. Even systems that are not differential-drive vehicles can be augmented with FLAIR thanks to its hierarchical approach by employing a lower-level controller that produces a high-level control law that acts as a differential-drive system, as demonstrated by the hexapod experiments in Section "Hexapod Results". While extending the concepts of FLAIR to more advanced robots, like humanoids or dexterous manipulators, remains an unexplored question, we believe this has great potential for future work.

The learned command-behaviour mapping model is constrained by the capabilities of the robot when a perturbation is experienced. In this section, we analyse these effects when the perturbation reaches its extreme values (full-loss of a track) and when the operator requests a behaviour located outside the feasible range.

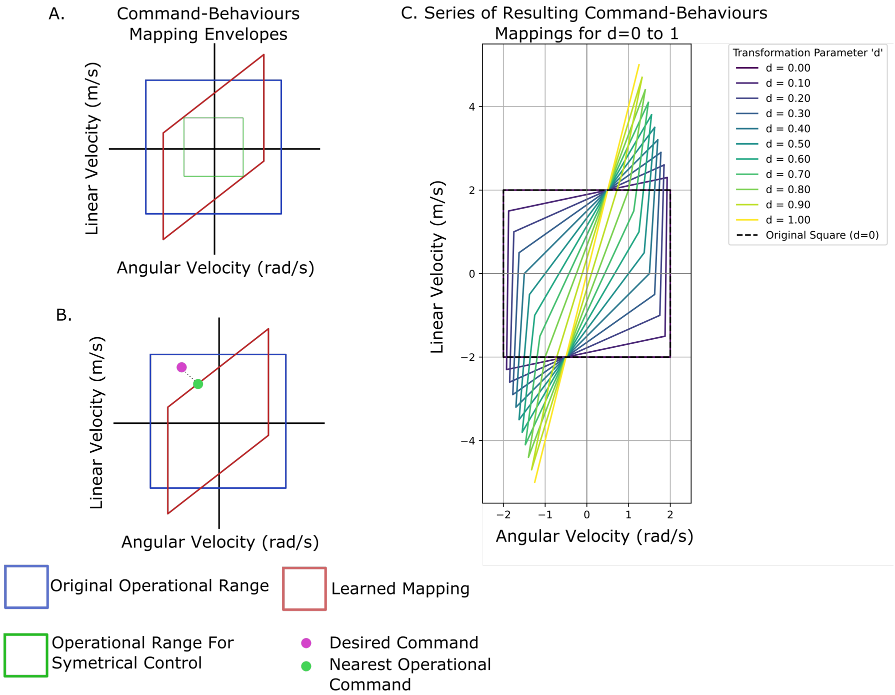

Fig. 2 FLAIR's Operational Range. (A) We show the operational range of the robot (blue) and the learned command-behaviour mapping (red). The operational range that allows the robot to execute commands in every axis is the green box. (B) We show that for the desired command (pink), FLAIR selects the nearest operational command available (green). (C) Full range of values for the global command-behaviours mapping between 0 and 1, showing how the operational range changes.

Let $d(\mathbf{s}) \in [-1, 1]$ represent the global commands-behaviour mapping dependent on state $\mathbf{s}$, as defined in the Methods Section - Global Adaptation To Perturbations. The modified control outputs $[v', \omega']$ are defined as a function of $d(\mathbf{s})$, where v and ω denote the original linear and angular velocities, respectively, given in Equation 8.

For a given value $d(\mathbf{s})$, we can compute the resulting operational range, i.e., how the model represents effects of the perturbation on the robot's ability to execute linear and angular displacements. Additionally, to provide a symmetrical operational range to the operator, the largest rectangle fitting within the learned mapping and centred around zero is used to process user intent. See Figure 2.A for an illustration.

When $d(\mathbf{s}) \to 1$ (i.e., when the Left Track suffers a complete failure), the resulting operational range becomes a line (see Figure 2.C). This implies that, in this configuration, the robot is unable to execute linear velocities without inducing an angular velocity. Its behaviour space is now limited to a single degree of freedom, which is consistent with the nature of the perturbation. A similar analysis can be performed for $d(\mathbf{s}) \to -1$, which results in the same conclusions, with the learned mappings being the symmetries (over the vertical axis) of Figure 2.C.

If the operator or the controller requests a behaviour situated outside the learned mapping, the nearest behaviour point within the mapping is selected instead (see

Figure 2.B). In practice, this means that a residual effect of the perturbation will remain and will be experienced by the operator. This typically occurs when the operator requests a behaviour that is outside the remaining capabilities of the robot.

The function $d(\mathbf{s})$ captures the impact of perturbations based on the robot's state. As described in the main text, FLAIR approximates this function as a polynomial function of the robot's state to form the global adaptation layer and augments it with a local approximation using a Gaussian Process.

These two functions (third-degree polynomial functions and Gaussian Process) both assume that the function to be approximated is smooth and continuous. While this assumption holds in all the scenarios considered in this paper, there may be certain types of perturbations that cannot be captured with a smooth and continuous function. In such a case, FLAIR will fit the closest continuous function to this perturbation and will remain inaccurate in the region of discontinuity. The driver will have to compensate for residuals left due to the discontinuity. Future extensions of FLAIR could be designed to handle discontinuities or complex functions, for instance, by using a global adaptation function based on piece-wise linear or polynomial functions with limited additional computational cost.

Supplementary Experimental Platform

Hardware

Our robot is an adapted PackBot [57] called GVR-Bot (https://www.army.mil/article/132285/GVR_Bots_to_Act_as_Research_Platforms/) that doesn't use any flippers. The robot is similar to most tracked vehicles and can only be controlled via two velocity commands (angular and linear velocities). To run our onboard computation, we are using an NVIDIA Jetson AGX Orin 32GB that we have mounted on top of the robot. We use two sensors on the robot, which we connect to the computer we have mounted; 1)A VN-100 vectornav IMU, and 2) a ZED2 camera providing the visual odometry fused with the IMU data.

For the automatic driver, we use a VICON motion capture system to guide the robot. The sole purpose of this system is to follow way-points by sending the correct commands and to provide a ground truth measure in the reported metrics. We do not use any data from the motion capture system in FLAIR.

Implementation and Use of Hardware Acceleration

The performance of FLAIR relies on its ability to run fully online on the embedded system and to learn new models in a matter of seconds. This requires both lightweight and fast computation abilities. To allow this, we optimise our code to efficiently utilise multiprocessing and GPU acceleration. The multiprocessing allows the different modules (data collection, model training and model inference) to run simultaneously, reducing delay in their execution. To leverage GPU acceleration, the model training of FLAIR is implemented using the Jax [55] library, which makes use of XLA compilation [58]. Our implementation of Gaussian process is based on the structure of the GPJax [59] library, redesigned and optimised for our usage. Our implementation

reduces the total model training and deployment time to less than 225ms (composed of $\approx$ 67ms to 215ms Gaussian Process Training + $\approx$ 9ms updating the controller commands and $\approx$ 3ms for data filtering). This is less than the median human reaction time for visual stimuli [60].

Detailed Perturbations

Each type of perturbation used in this paper is simulated as follows:

- Static perturbations: we simulate this perturbation by scaling the requested tracks' velocity with scaling factors $d_{\text{left}}, d_{\text{right}} \in [0, 1]$ that remain constant throughout the run.
- Dynamic perturbations: we apply a perturbation with scaling factors $d_{\text{left}}(t), d_{\text{right}}(t) \in [0, 1]$ but change the values of these factors across the deployment period.
- State-Dependent perturbations: the scaling factor $d_{\text{left}}(s), d_{\text{right}}(s) \in [0, 1]$ changes with state s of the robot, such as its speed or orientation.

Our robot controller allows for two commands, the linear velocity v_x and the rotation speed ω_z. To simulate the perturbation on a single track, we use the differential drive kinematics of the robot to transform v_x and ω_z into track speeds t_{left} and t_{right}. To do the conversion, we use the width of the robot h and the radius of the wheels $radius$. The scaling factor $d = (d_{\text{left}}, d_{\text{right}})$ is applied to the commands that will be executed by the robot and influences the commanded track speeds t_{left} and t_{right}:

$$t_{\text{left}} = \frac{v_x - \omega_z \frac{h}{2}}{\text{radius}} \tag{10}$$

$$t_{\text{right}} = \frac{v_x + \omega_z \frac{h}{2}}{\text{radius}} \tag{11}$$

and finally, we can then scale the track speeds before transforming them back into perturbed velocities v'_x and ω'_z:

$$t'_{\text{left}} = t_{\text{left}} * d_{\text{left}}$$

$$t'_{\text{right}} = t_{\text{right}} * d_{\text{right}}$$

$$v'_x = 0.5(t'_{\text{left}} + t'_{\text{right}}) * \text{radius} \tag{12}$$

$$\omega'_z = (t'_{\text{right}} - t'_{\text{left}}) \frac{\text{radius}}{h} \tag{13}$$

Detailed Experimental Circuits

To evaluate FLAIR's capacity to restore operability when faced with perturbations during deployment, we designed 3 circuits that the robot needs to complete, illustrated in Figure 3. The goal is for an operator to successfully drive around these circuits, which consist of ramps and a range of simulated perturbations that need to be navigated.

Circuit **1**: We first test FLAIR on a chicane illustrated in Figure 3.A, with static and dynamic perturbations. We run separate experiments to evaluate both these conditions. In the former, we apply a static perturbation on the right track with factor $d_{\text{right}} = 0.7$, meaning this track is only working at 70% of its capacity. In the latter, the scaling factor d_{right} alternates between 0.6 and 0.8 at pre-defined waypoints on the chicane circuit. To ablate the effect of perturbations, we include a third set of experiments on this chicane circuit by testing how the performance changes with the intensity of the static perturbations (from $d_{\text{right}} = 0.5$ to $d_{\text{right}} = 1.0$).
Circuit **2**: The second circuit (Figure 3.B) is split into two sections. The operator is expected to perform 1 loop on the ramp while having a static perturbation on the right track with a factor $d_{\text{right}} = 0.7$ and $d_{\text{left}} = 1.0$. This perturbation scales down the speed of the right track motor to 70% of its nominal speed. This induced perturbation adds to the shifted CoM of our robot as well as the slippage that naturally occurs on the ramp, and makes it very challenging to turn while gravity pulls the robot toward the bottom of the ramp.

The second section of Circuit 2 requires navigating 4 loops while impacted by an artificial wind, which we simulate as a state-dependent perturbation (see Figure 3.B bottom). The assumption regarding the wind is that it is continuously blowing in a fixed direction. This makes the perturbation state-dependent for the robot since the sail of the robot would have a different area of attack for the wind, directly influencing the force that is applied to the robot. In our closed room, the wind is achieved by varying the scaling factors $d_{\text{left}}(s)$ and $d_{\text{right}}(s)$, which are dependent on the state $s = yaw$ of the robot's orientation. This means that if the wind is stronger (i.e. perpendicular to the robot's sail), the scaling is the highest. We decrease the scaling linearly with the rotation and disable the scaling if the robot is parallel to the wind. Importantly, FLAIR does not have direct access to the underlying function but must learn the perturbations solely from the data it collects. A single run of Circuit 2 includes the robot going up the ramp, performing 1 circle there with static perturbation, then going down the ramp and performing 4 circles on the floor with the state-dependent wind perturbation.

This circuit has been intentionally designed to include the transition between two different types of perturbation to demonstrate that the FLAIR system is not impacted by this shift in operating conditions and perturbation types.
Circuit **3**: We test the robustness of FLAIR on Circuit 3 (Figure 3.C) with different elements on the circuit. The wedged bump induces a change in the inclination of the robot, as well as a shock when passing its top that produces vibrations and thus noisy sensor data. The slippery surface leads to friction changes that act as an additional natural perturbation, and it can be slightly dragged by the tracks of the robot, leading to complex changes in the dynamics. Finally, the metal grid is slightly thicker than the track of the robot, lifting the side of the robot when driving on it, while also inducing a lot of vibration and sensor noise for each new bar the robot crosses. These additional obstacles are added to test the robustness of FLAIR when encountering elements that add noisy local data that are uncharacteristic of the underlying global perturbation. This specifically evaluates the capabilities of FLAIR

to filter out noise and stochasticity caused by vibrations and collisions, and naturally-occurring perturbations such as friction changes, and still make accurate predictions of the perturbations. Ensuring that FLAIR is robust to such unreliable data is crucial to allow its deployment in real-world scenarios. As done in Circuit 2, we consider a static perturbation on the right track with a factor $d_{\text{right}} = 0.7$. The setup of Circuit 3 is subject to variability from one run to another as objects and obstacles can be slightly moved and might not impact all the runs consistently. Hence, the results for this section are expected to display greater variation than for the other controlled experiments.

Automatic Driver

For the integrated circuit, we used a simple algorithm to track the way-points that we defined beforehand, similarly to Caluwaerts et al. [61]. We use a VICON motion capture system with 10 cameras to track the robot's position and orientation with respect to the waypoints. The way-point tracker sends an angular and linear velocity as high-level commands:

$$
\begin{aligned}
v_x &= \begin{cases} 0.5 * K, & \text{if } -\pi/4 < \beta < \pi/4 \\ 0, & \text{else} \end{cases} \\
\omega_z &= \beta
\end{aligned}
$$

with K being the distance to the way-point and β the heading of the robot with respect to the way-point.

Replications

To provide representative comparisons, we do 20 replications per method and per experiment on each circuit.

Supplementary Results

Detailed Metrics Computation

This section details the computation of the metrics used in the experimental section. Importantly, the Tracking Error relies on sensor readings from our VICON motion capture system for the ground-truth, while FLAIR uses onboard sensor readings, that are intrinsically more error-prone.
Completion Time: Total time in seconds taken by each replication to complete the section. This is computed by the automatic driver using the readings from the VICON motion capture system. The robot might be initialised in a slightly different position from one run to another, depending on the chaining of replications. Thus, at the start of every replication, the automatic driver is tasked to reset the robot to a default initial position for the section, and the start time is only acquired when this default position is reached. The end time is taken at the time when the robot reaches the final

Perturbation		Static	Dynamic	Static	State-Dep.	Static	
Circuit		1	1	2	2	3	
Section		Chicane Static	Chicane Dynamic	Ramp	Wind	Robust.	Median
(A) Completion Time increase	Driver	34.2%	63.5%	53.3%	46.3%	57.2%	53.3%
	Driver + FLAIR	9.5%	21.8%	3.1%	8.9%	17.2%	9.5%
	Driver + FLAIR [No Perturb]	0.3%	0.3%	−1.7%	0.1%	7.2%	0.3%
	LQR	14.3%	32.1%	15.3%	29.6%	18.9%	18.9%
	$\mathcal{L}_1$	7.4%	24.5%	20.8%	22.4%	10.3%	20.8%
(B) FLAIR Time Completion Reduction		72.2%	65.7%	94.1%	80.7%	69.9%	72.2%

Table 3 Summary of FLAIR's Performances according to Completion Time. Overhead with respect to the "Driver [No Perturbation]" baseline in term of circuit Completion Time (A). We also quantify the Time Completion Reduction by FLAIR as the reduction in increased time (B) for "Driver + FLAIR" with respect to the "Driver" (100% means the overhead in time or error is gone). For all metrics, we compare the medians over 20 replications.

target of the section. The Completion Time corresponds to the difference between the end and start time, in seconds.
Tracking Error: Error between the command sent to the robot and the behaviour it executes. This is computed using the command sent by the automatic driver and the behaviour read by the VICON motion capture system. As it takes some time for the robot to implement a given command, before computing the error, we synchronise the command and sensor readings. We dynamically compute a command-sensor delay for each command sent by the automatic driver using cross-correlation [62, 63] using a buffer of 3 seconds of data around the considered command timestamp (1/4 of the data before the timestamp and 3/4 after). This matching step constitutes command-sensor pairs containing the command sent by the automatic driver and the resulting effect in sensor reading. For each pair, we compute the timestep Tracking Error as the norm of the distance between command and sensor readings. For each replication, we return the final Tracking Error as the median of the Tracking Error over the section.

Completion Time increase and Tracking Error increase: As the Completion Time and Tracking Error metrics are hard to interpret when provided as absolute values, we provide the results of a "Driver [No Perturbation]" baseline. This baseline does not consider any artificially induced perturbation and estimates how well the robot would do on the circuit when operable. Thus, we propose to quantify the percentage increase with respect to this baseline for every other algorithm. This gives the Completion Time increase and Tracking Error increase metrics, reported in our results. For any metrics M and for any algorithms alg, the metric M increase is defined as follows:

$$M_{increase}(\text{alg}) = \frac{M(\text{alg}) - M(\text{Driver [No Perturbation]})}{M(\text{Driver [No Perturbation]})} * 100$$

FLAIR operability reconstitution: The operability reconstitution can be computed for both the Completion Time and the Tracking Error. It quantifies the proportion of operability that has been recovered by FLAIR according to the chosen metric. For a metric M and the corresponding $M_{increase}$ metric, it is defined as follows:

$$M_{reconstitution} = \left(1 - \frac{M_{increase}(\text{Driver + FLAIR})}{M_{increase}(\text{Driver})}\right) * 100$$

Update time for FLAIR: total time in ms taken to update the Command-aware Buffer, apply the Soft Outlier Rejection mechanism, train the global and local models of the effect of perturbation, and deploy this new model for inference.

Additional Results for Completion Time

We provide in Table 3 the Completion Time increase and Operability Reconstitution according to Completion Time. The same metrics are provided for Tracking Error in the main paper.

Statistical Analysis

To highlight the statistical significance of our results, we provide in Table 4 the p-values of the comparison of the driver with and without FLAIR in each section.

Detailed Results For Chicane with Increasing Perturbation Strength

We provide in Figure 3 the detailed boxplot comparison for the Chicane Static with increasing perturbation strength, similar to the boxplot for the different Circuits provided in the main paper.

Results

We run the automatic driver on the three circuits without ("Driver") and with FLAIR ("Driver + FLAIR"). We also run the automatic driver on the three circuits without any simulated perturbation to get a baseline to compare our metrics to. This estimates how well the robot should do under full operability without any perturbations.

Perturbation	Static	Dynamic	Static	State-Dep.	Static
Circuit	1	1	2	2	3
Section	Chicane Static	Chicane Dynamic	Ramp	Wind	Robustness
Completion Time Driver	$6.3e-08$	$6.3e-08$	$1.1e-07$	$4.9e-07$	$3.0e-08$
Tracking Error Driver	$6.3e-08$	$6.3e-08$	$6.3e-08$	$6.3e-08$	$3.0e-08$
Completion Time LQR	$5.9e-03$	$7.9e-06$	$9.2e-06$	$4.6e-05$	$9.8e-01$
Tracking Error LQR	$1.7e-05$	$3.7e-07$	$3.7e-06$	$1.2e-03$	$5.6e-04$
Completion Time $\mathcal{L}_1$	$1.3e-01$	$2.1e-01$	$2.7e-01$	$3.5e-03$	$2.3e-03$
Tracking Error $\mathcal{L}_1$	$2.6e-05$	$3.3e-06$	$7.2e-07$	$6.3e-08$	$3.3e-05$

Table 4 Statistical analysis. p-values computed using Wilcoxon Rank test for the comparison of (top) the "Driver" and "Driver + FLAIR" results for each section, and (middle) the "LQR" or (bottom) $\mathcal{L}_1$ and "Driver + FLAIR" results for each section.

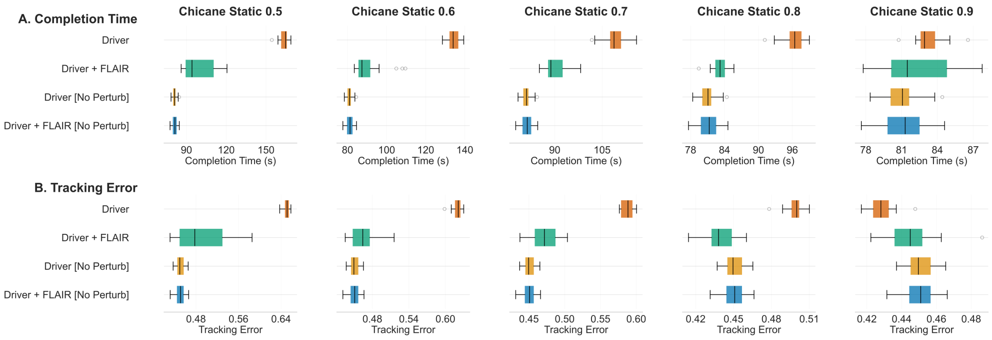

Fig. 3 FLAIR's Performance on the Chicane with increasing perturbation strength. We compare FLAIR against baselines to show the benefits on (A) circuit Completion Time and (B) Tracking Error for the Chicane Static with increasing perturbation strength (strength increases from left to right). In the box-plots, the center line represents the median, the box limits denote the upper and lower quartiles, the whiskers extend to 1.5x the interquartile range, and individual points represent outliers.

We denote this baseline as "Driver [No Perturbation]". Finally, to measure the overhead caused by our method, we also run the automatic driver with FLAIR in the no perturbation setup, denoted "Driver + FLAIR [No Perturbation]".

For the Chicane Static and Dynamic section (Circuit 1 in Figure 3.A), we observe that on the trajectory plots, "Driver + FLAIR" stays much closer to the reference trajectory ("Driver [No Perturbation]"; black line), whereas the "Driver" deviates from the trajectory in a lot of cases. As expected, FLAIR needs a little bit of adaptation time on the straight line at the bottom of the Chicane in the first lap, but follows the reference trajectory in the second lap. As mentioned earlier, the robot does not have a uniform weight distribution due to its batteries, leading it to turn right faster than left. The results indicate that FLAIR compensates for this natural perturbation

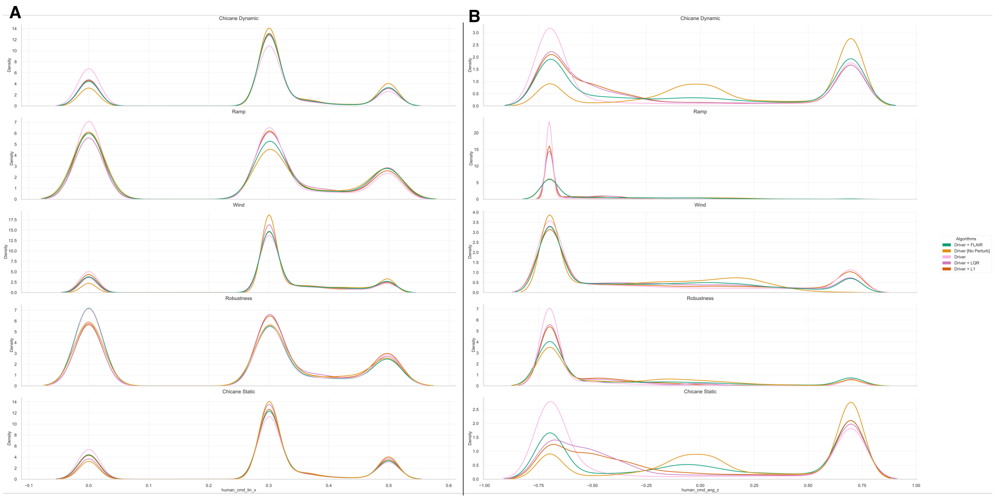

Fig. 4 $\mathcal{L}_1$ Adaptive Control baseline detailed analysis. For each Circuit, we plot the commands used by the driver to get to each point. The "Driver" baseline constitutes the commands by a damaged vehicle, while the "Driver [No Perturb]" is the best case possible. We can see that in most cases "Driver + FLAIR" is closer to the "Driver [No Perturb]" baseline in terms of commands, which suggests that the driving behaviour is more similar to the non-perturbed case. In contrast, we see that the baselines sometimes use very large commands, showing how the robot could achieve a shorter Circuit Completion Time by driving faster while ignoring the commands sent by the driver. This is directly reflected by the Tracking Error metric. We show the Kernel Density Estimation (KDE) plots of the angular commands in (A) and of the linear velocity commands in (B).

even when no additional simulated perturbation is considered ("Driver + FLAIR [No Perturbation]"), resulting in a negative overhead of 0.3% in Completion Time and 0.7% in Tracking Error.

For the Chicane Dynamic section (Circuit 1 in Figure 3.A), the perturbation is modified multiple times while driving around the circuit, requiring FLAIR to successfully detect these changes and quickly learn a new model of their effects to be able to compensate. FLAIR proves able to successfully handle these dynamic perturbations, recovering 65.7% of operability in Completion Time and 56.6% in Tracking Error. It is important to note that these metrics encompass the additional time and error spent detecting and learning the new perturbation effects four times per run. These additional learning phases are also visible on the trajectory plots on the left and top right of the Chicane track. The comparison of the "Driver + FLAIR" results for the Chicane Static and Dynamic indicates that these three additional learning phases only induce a 12.3% Completion Time increase, highlighting the fast-learning abilities of FLAIR.

For the Ramp section (Circuit 2 in Figure 3.B), the combination of the perturbations makes it hard to turn at the top of the ramp, causing the robot to systematically get stuck there for some time, as visible on the trajectory plots from the longer time spent between way-points in this area (darker colour). Getting out of this blocking point faster requires a strong compensation on the right track in addition to the static damage compensation.

C. Trajectories

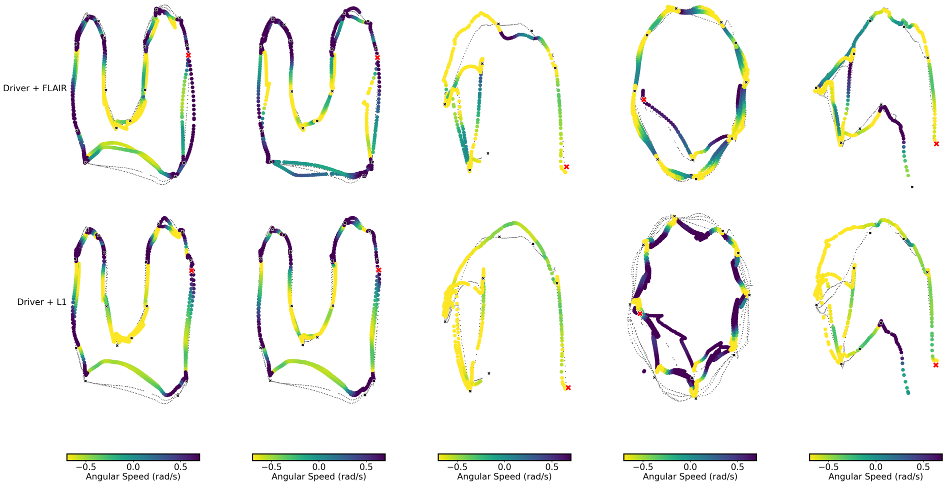

Fig. 5 Intensity of Angular Velocity Command. We display trajectories of one randomly-picked replication of "Driver + FLAIR" and "Driver + L1". The colour indicates the angular velocity command (the darker or the lighter, the larger the command). We see that L1 requires the driver to use larger driver commands to make the robot move, indicating that the driver is less in control.

Despite this complexity, while the driver alone ("Driver") requires double the time to complete the Ramp section compared to the unperturbed case ("Driver [No Perturbation]"; 53.3%), the driver assisted by FLAIR ("Driver + FLAIR") only requires a 3.1% increase. The results are similar for the Tracking Error, where the "Driver" alone has a 29.8% error increase, while "Driver + FLAIR" only has a −0.3% increase.

The Wind section (Circuit 2 in Figure 3.B) highlights the ability of FLAIR to generalise its knowledge and learn a global model of the effect of perturbations, while it hasn't yet encountered all the possible orientations. Without FLAIR, the operator needs 46.3% more time to complete the Wind section but once FLAIR is active, the operator is able to come within 8.9% of the baseline performance, recovering 80.7% of operability in time and 74.7% in error. This discrepancy is also visible on the trajectory plots, where "Driver" takes longer at each step (darker colour) due to the Wind effect. This result includes the time and error spent detecting the change, resetting and adapting again to the new perturbations.

Additionally, we provide in Figure 4 and Figure 5 detailed analysis of our $\mathcal{L}_1$ Adaptive Control baseline. These analyses highlight how the $\mathcal{L}_1$ baseline is managing to finish the Circuits with lower Completion time but higher Tracking Error.

Supplementary Methods

Parameters for Hierarchical Online Adaptation

The Gaussian process we use for our model f has the following parameters: the values for the length-scale parameters are $l = (0.69437875, 0.56324503, 10.0)$, the observation

noise is 0.01750011, and the variance is 0.04772636. These values are determined by maximising the log-marginal-likelihood [64] using data collected while the robot operates under non-perturbed conditions. To collect these data points, we assume the robot is initially operated under non-perturbed conditions for a few seconds using the pipeline described in this paper. After these few seconds of initial operation, we freeze the parameters throughout all the experiments to improve the speed of the model.

Additional Details on Dataset Creation

Synchronisation in Data Point Creation: For the Data Point Creation, the commands from the controller and the behaviour from the sensors first need to be synchronised and matched. This is done dynamically based on a buffer containing T seconds of past commands $(\mathbf{a_i})_{0\leq i\leq A_T}$ and behaviours $(\mathbf{b_i})_{0\leq i\leq B_T}$. The synchronised data $(\mathbf{b_i})_{-d\leq i\leq B_T-d}$ is computed using cross-correlation [62, 63], denoted $\star$:

$$d = \underset{0\leq d'\leq B_T}{\arg\max} \left((\mathbf{a_i})_{0\leq i\leq A_T} \star (\mathbf{b_i})_{-d'\leq i\leq B_T-d'}\right)$$

By computing this matching dynamically instead of using a fixed or pre-defined value, our system accounts for the changes in dynamics caused by the environment and by the perturbations themselves [62, 63]. In summary, this first step matches the command and sensor signal.

Filtering in Data Point Creation: After creating the data points, one important issue remains; due to the inertia of the system, some commands might not have the time to be fully executed before the command is changed by the controller. This happens when the command frequency is higher than the frequency of response of the system. For example, if the robot is at a stop and the controller requires a linear speed of $1m.s^{-1}$ and immediately after requires a speed of $0.5m.s^{-1}$, the robot would not have the time to reach the $1m.s^{-1}$ and would likely stabilise at $0.5m.s^{-1}$. The Data Point Creation proposes an additional filter for this case that simply does not return any data points when the command value has changed too drastically. In other words, this filter gets rid of data points corresponding to transition states that do not carry meaningful information for the perturbation model.

Command-aware buffer: The Command-aware buffer, detailed in Materials and Methods, uses a double FIFO mechanism: at the level of the cell and at the level of the grid as a whole. Here, we aim to expand on the need for these two mechanisms. Without the grid-wide FIFO, while cell-FIFO mechanisms guarantee the coverage of the command space, it introduces a trade-off between the grid dimensions and the total size of the dataset. For example, drastically reducing the total size of the dataset would require a loss in the precision of the grid discretisation, which might lead to other side effects. Thus, to avoid this trade-off while keeping the dataset size reasonable, our proposed buffer also has a total size that is enforced by removing the oldest data points in the grid, regardless of their cells. To summarise, a data point already in the grid might either get replaced by a newer one if it is the oldest data point currently in its cell, or it might get deleted from the grid if it is among the oldest data points overall.

Effect of Dimensionality of Data on Learning Speed

First, increasing the dimensionality of training data for the GP has a complexity of $\mathcal{O}(n)$ with the number of dimensions n, while the dataset size is usually $\mathcal{O}(n^3)$ with the number of training datapoints n. Therefore, FLAIR should be able to achieve rapid adaptation even in larger control input spaces, but will be slowed down by an important increase in training size. To this end, the Dataset Creation module is specifically designed to build a dataset that is both compact and highly informative, with the total number of datapoints always kept fixed at 1000. This design choice ensures that model training remains computationally efficient and feasible in real-time settings. Second, FLAIR is designed such that model training runs concurrently and in parallel with all other processes. Consequently, even if training becomes slightly slower due to increased computational demands, it would only reduce the frequency of model updates rather than hindering the robot's overall operation.

References

[1] Kelly, M.: An introduction to trajectory optimization: How to do your own direct collocation. SIAM Rev. **59**, 849–904 (2017)

[2] Nascimento, T.P., Dórea, C.E., Gonçalves, L.M.G.: Nonholonomic mobile robots' trajectory tracking model predictive control: a survey. Robotica **36**(5), 676–696 (2018)

[3] Di Carlo, J., Wensing, P.M., Katz, B., Bledt, G., Kim, S.: Dynamic locomotion in the mit cheetah 3 through convex model-predictive control. In: 2018 IEEE/RSJ International Conference on Intelligent Robots and Systems (IROS), pp. 1–9 (2018). IEEE

[4] Pankert, J., Hutter, M.: Perceptive Model Predictive Control for Continuous Mobile Manipulation. IEEE Robotics and Automation Letters **5**(4), 6177–6184 (2020) https://doi.org/10.1109/LRA.2020.3010721

[5] Jenelten, F., Hwangbo, J., Tresoldi, F., Bellicoso, C.D., Hutter, M.: Dynamic locomotion on slippery ground. IEEE Robotics and Automation Letters **4**(4), 4170–4176 (2019)

[6] Bledt, G., Wensing, P.M., Ingersoll, S., Kim, S.: Contact model fusion for event-based locomotion in unstructured terrains. In: 2018 IEEE International Conference on Robotics and Automation (ICRA), pp. 4399–4406 (2018). IEEE

[7] O'Connell, M., Shi, G., Shi, X., Azizzadenesheli, K., Anandkumar, A., Yue, Y., Chung, S.-J.: Neural-fly enables rapid learning for agile flight in strong winds. Science Robotics **7**(66), 6597 (2022)

[8] Sutton, R.S., Barto, A.G.: Reinforcement Learning: An Introduction. MIT press, ??? (2018)

[9] Hwangbo, J., Lee, J., Dosovitskiy, A., Bellicoso, D., Tsounis, V., Koltun, V., Hutter, M.: Learning agile and dynamic motor skills for legged robots. Science Robotics **4**(26), 5872 (2019)

[10] Lee, J., Hwangbo, J., Wellhausen, L., Koltun, V., Hutter, M.: Learning quadrupedal locomotion over challenging terrain. Science robotics **5**(47), 5986 (2020)

[11] Margolis, G.B., Agrawal, P.: Walk these ways: Tuning robot control for generalization with multiplicity of behavior. In: Conference on Robot Learning, pp. 22–31 (2023). PMLR

[12] Tobin, J., Fong, R., Ray, A., Schneider, J., Zaremba, W., Abbeel, P.: Domain randomization for transferring deep neural networks from simulation to the real world. In: 2017 IEEE/RSJ International Conference on Intelligent Robots and Systems (IROS), pp. 23–30 (2017). IEEE

[13] Sadeghi, F., Levine, S.: CAD2RL: real single-image flight without a single real image. In: Amato, N.M., Srinivasa, S.S., Ayanian, N., Kuindersma, S. (eds.) Robotics: Science and Systems XIII, Massachusetts Institute of Technology, Cambridge, Massachusetts, USA, July 12-16, 2017 (2017). https://doi.org/10.15607/RSS.2017.XIII.034 . http://www.roboticsproceedings.org/rss13/p34.html

[14] Peng, X.B., Andrychowicz, M., Zaremba, W., Abbeel, P.: Sim-to-real transfer of robotic control with dynamics randomization. In: 2018 IEEE International Conference on Robotics and Automation (ICRA), pp. 3803–3810 (2018). IEEE

[15] Akkaya, I., Andrychowicz, M., Chociej, M., Litwin, M., McGrew, B., Petron, A., Paino, A., Plappert, M., Powell, G., Ribas, R., et al.: Solving rubik's cube with a robot hand. arXiv preprint arXiv:1910.07113 (2019)

[16] Makoviychuk, V., Wawrzyniak, L., Guo, Y., Lu, M., Storey, K., Macklin, M., Hoeller, D., Rudin, N., Allshire, A., Handa, A., State, G.: Isaac gym: High performance gpu-based physics simulation for robot learning. CoRR **abs/2108.10470** (2021) 2108.10470

[17] Freeman, C.D., Frey, E., Raichuk, A., Girgin, S., Mordatch, I., Bachem, O.: Brax - a differentiable physics engine for large scale rigid body simulation (2021)

[18] Khetarpal, K., Riemer, M., Rish, I., Precup, D.: Towards continual reinforcement learning: A review and perspectives. Journal of Artificial Intelligence Research **75**, 1401–1476 (2022)

[19] Abel, D., Barreto, A., Van Roy, B., Precup, D., Hasselt, H.P., Singh, S.: A definition of continual reinforcement learning. Advances in Neural Information Processing Systems **36**, 50377–50407 (2023)

[20] Finn, C., Abbeel, P., Levine, S.: Model-agnostic meta-learning for fast adaptation of deep networks. In: International Conference on Machine Learning, pp. 1126–1135 (2017). PMLR

[21] Beck, J., Vuorio, R., Liu, E.Z., Xiong, Z., Zintgraf, L., Finn, C., Whiteson, S., *et al.*: A tutorial on meta-reinforcement learning. Foundations and Trends® in Machine Learning **18**(2-3), 224–384 (2025)

[22] Duan, Y., Schulman, J., Chen, X., Bartlett, P.L., Sutskever, I., Abbeel, P.: Rl ^{2}: Fast reinforcement learning via slow reinforcement learning. arXiv preprint arXiv:1611.02779 (2016)

[23] Silver, T., Allen, K., Tenenbaum, J., Kaelbling, L.: Residual policy learning. arXiv preprint arXiv:1812.06298 (2018)

[24] Johannink, T., Bahl, S., Nair, A., Luo, J., Kumar, A., Loskyll, M., Ojea, J.A., Solowjow, E., Levine, S.: Residual reinforcement learning for robot control. In: 2019 International Conference on Robotics and Automation (ICRA), pp. 6023–6029 (2019). IEEE

[25] Dohare, S., Hernandez-Garcia, J.F., Lan, Q., Rahman, P., Mahmood, A.R., Sutton, R.S.: Loss of plasticity in deep continual learning. Nature **632**(8026), 768–774 (2024)

[26] Bongard, J., Zykov, V., Lipson, H.: Resilient machines through continuous self-modeling. Science **314**(5802), 1118–1121 (2006) https://doi.org/10.1126/science.1133687 https://www.science.org/doi/pdf/10.1126/science.1133687

[27] Chen, B., Kwiatkowski, R., Vondrick, C., Lipson, H.: Fully body visual self-modeling of robot morphologies. Science Robotics **7**(68), 1944 (2022)

[28] Nygaard, T.F., Martin, C.P., Torresen, J., Glette, K., Howard, D.: Real-world embodied ai through a morphologically adaptive quadruped robot. Nature Machine Intelligence **3**(5), 410–419 (2021)

[29] Cully, A., Clune, J., Tarapore, D., Mouret, J.-B.: Robots that can adapt like animals. Nature **521**(7553), 503–507 (2015)

[30] Pugh, J.K., Soros, L.B., Stanley, K.O.: Quality diversity: A new frontier for evolutionary computation. Frontiers in Robotics and AI **3**, 40 (2016)

[31] Chatzilygeroudis, K., Cully, A., Vassiliades, V., Mouret, J.-B.: Quality-diversity optimization: a novel branch of stochastic optimization. In: Black Box Optimization, Machine Learning, and No-Free Lunch Theorems, pp. 109–135. Springer, ??? (2021)

[32] Chatzilygeroudis, K., Vassiliades, V., Mouret, J.-B.: Reset-free trial-and-error

learning for robot damage recovery. Robotics and Autonomous Systems **100**, 236–250 (2018)

[33] Allard, M., Smith, S.C., Chatzilygeroudis, K., Lim, B., Cully, A.: Online damage recovery for physical robots with hierarchical quality-diversity. ACM Transactions on Evolutionary Learning **3**(2), 1–23 (2023)

[34] Milecki, A., Nowak, P.: Review of fault-tolerant control systems used in robotic manipulators. Applied Sciences **13**(4) (2023) https://doi.org/10.3390/app13042675

[35] Baioumy, M., Pezzato, C., Ferrari, R., Corbato, C.H., Hawes, N.: Fault-tolerant control of robot manipulators with sensory faults using unbiased active inference. In: 2021 European Control Conference (ECC), pp. 1119–1125 (2021). IEEE

[36] Elsayed, A.M., Elshalakani, M., Hammad, S.A., Maged, S.A.: Decentralized fault-tolerant control of multi-mobile robot system addressing lidar sensor faults. Scientific Reports **14**(1), 25713 (2024)

[37] Pio-Lopez, L., Nizard, A., Friston, K., Pezzulo, G.: Active inference and robot control: a case study. Journal of The Royal Society Interface **13**(122), 20160616 (2016)

[38] Baioumy, M., Pezzato, C., Corbato, C.H., Hawes, N., Ferrari, R.: Towards stochastic fault-tolerant control using precision learning and active inference. In: Joint European Conference on Machine Learning and Knowledge Discovery in Databases, pp. 681–691 (2021). Springer

[39] Pezzato, C., Ferrari, R., Corbato, C.H.: A novel adaptive controller for robot manipulators based on active inference. IEEE Robotics and Automation Letters **5**(2), 2973–2980 (2020)

[40] Baioumy, M., Duckworth, P., Lacerda, B., Hawes, N.: Active inference for integrated state-estimation, control, and learning. In: 2021 IEEE International Conference on Robotics and Automation (ICRA), pp. 4665–4671 (2021). IEEE

[41] Baioumy, M., Pezzato, C., Ferrari, R., Hawes, N.: Unbiased active inference for classical control. In: 2022 IEEE/RSJ International Conference on Intelligent Robots and Systems (IROS), pp. 12787–12794 (2022). IEEE

[42] Shi, G., Shi, X., O'Connell, M., Yu, R., Azizzadenesheli, K., Anandkumar, A., Yue, Y., Chung, S.-J.: Neural lander: Stable drone landing control using learned dynamics. In: 2019 International Conference on Robotics and Automation (icra), pp. 9784–9790 (2019). IEEE

[43] Li, Q., Qian, J., Zhu, Z., Bao, X., Helwa, M.K., Schoellig, A.P.: Deep neural networks for improved, impromptu trajectory tracking of quadrotors. In: 2017

IEEE International Conference on Robotics and Automation (ICRA), pp. 5183–5189 (2017). IEEE

[44] Hovakimyan, N., Cao, C., Kharisov, E., Xargay, E., Gregory, I.M.: L1 adaptive control for safety-critical systems. IEEE Control Systems Magazine **31**(5), 54–104 (2011) https://doi.org/10.1109/MCS.2011.941961

[45] Kalman, R.E., *et al.*: Contributions to the theory of optimal control. Bol. soc. mat. mexicana **5**(2), 102–119 (1960)

[46] Lehtomaki, N., Sandell, N., Athans, M.: Robustness results in linear-quadratic gaussian based multivariable control designs. IEEE Transactions on Automatic Control **26**(1), 75–93 (1981) https://doi.org/10.1109/TAC.1981.1102565

[47] Shaiju, A.J., Petersen, I.R.: Formulas for discrete time lqr, lqg, leqg and minimax lqg optimal control problems. IFAC Proceedings Volumes **41**(2), 8773–8778 (2008) https://doi.org/10.3182/20080706-5-KR-1001.01483 . 17th IFAC World Congress

[48] Starin, S.R., Yedavalli, R.K., Sparks, A.G.: Design of a lqr controller of reduced inputs for multiple spacecraft formation flying. In: Proceedings of the 2001 American Control Conference. (Cat. No.01CH37148), vol. 2, pp. 1327–13322 (2001). https://doi.org/10.1109/ACC.2001.945908

[49] Feng, X., Liu, S., Yuan, Q., Xiao, J., Zhao, D.: Research on wheel-legged robot based on lqr and adrc. Scientific reports **13**(1), 15122 (2023)

[50] Hasan, S.K., Dhingra, A.: Developing a linear quadratic regulator for human lower extremity exoskeleton robot. Journal of Mechatronics and Robotics **6**, 28–46 (2022) https://doi.org/10.3844/jmrsp.2022.28.46

[51] Xin, G., Xin, S., Cebe, O., Pollayil, M.J., Angelini, F., Garabini, M., Vijayakumar, S., Mistry, M.: Robust footstep planning and lqr control for dynamic quadrupedal locomotion. IEEE Robotics and Automation Letters **6**(3), 4488–4495 (2021)

[52] Todorov, E., Li, W.: A generalized iterative lqg method for locally-optimal feedback control of constrained nonlinear stochastic systems. In: Proceedings of the 2005, American Control Conference, 2005., pp. 300–306 (2005). IEEE

[53] Fujimoto, S., Hoof, H., Meger, D.: Addressing function approximation error in actor-critic methods. In: International Conference on Machine Learning, pp. 1587–1596 (2018). PMLR

[54] Huang, S., Dossa, R.F.J., Ye, C., Braga, J., Chakraborty, D., Mehta, K., Araújo, J.G.M.: Cleanrl: High-quality single-file implementations of deep reinforcement learning algorithms. Journal of Machine Learning Research **23**(274), 1–18 (2022)

[55] Bradbury, J., Frostig, R., Hawkins, P., Johnson, M.J., Leary, C., Maclaurin, D., Necula, G., Paszke, A., VanderPlas, J., Wanderman-Milne, S., Zhang, Q.: JAX: composable transformations of Python+NumPy programs (2018)

[56] Mouret, J.-B., Clune, J.: Illuminating search spaces by mapping elites. arXiv preprint arXiv:1504.04909 (2015)

[57] Yamauchi, B.M.: Packbot: a versatile platform for military robotics. In: Unmanned Ground Vehicle Technology VI, vol. 5422, pp. 228–237 (2004). SPIE

[58] Sabne, A.: XLA : Compiling Machine Learning for Peak Performance (2020)

[59] Pinder, T., Dodd, D.: Gpjax: A gaussian process framework in jax. Journal of Open Source Software **7**(75), 4455 (2022) https://doi.org/10.21105/joss.04455

[60] Jain, A., Bansal, R., Kumar, A., Singh, K.: A comparative study of visual and auditory reaction times on the basis of gender and physical activity levels of medical first year students. International journal of applied and basic medical research **5**(2), 124–127 (2015)

[61] Caluwaerts, K., Iscen, A., Kew, J.C., Yu, W., Zhang, T., Freeman, D., Lee, K.-H., Lee, L., Saliceti, S., Zhuang, V., et al.: Barkour: Benchmarking animal-level agility with quadruped robots. arXiv preprint arXiv:2305.14654 (2023)

[62] Ianniello, J.: Time delay estimation via cross-correlation in the presence of large estimation errors. IEEE Transactions on Acoustics, Speech, and Signal Processing **30**(6), 998–1003 (1982)

[63] Benesty, J., Chen, J., Huang, Y.: Time-delay estimation via linear interpolation and cross correlation. IEEE Transactions on speech and audio processing **12**(5), 509–519 (2004)

[64] Neal, R.M.: Bayesian Learning for Neural Networks vol. 118. Springer, ??? (2012)
