# Supplementary material for: Getting robots back on track by reconstituting control in unexpected situations with online learning
Source: Nat Commun. 2026 Mar 9;17:3715. doi: 10.1038/s41467-026-70256-y (PMC13102915; doi:10.1038/s41467-026-70256-y)
Supplement: Supplementary file 2 — Description of Additional Supplementary Information [file 41467_2026_70256_MOESM2_ESM.pdf]

Description of Additional Supplementary Files

File Name: Supplementary Movie 1
Description: Explanation of the algorithm and showcase of the algorithm being deployed on a physical robot.
